# Kinetic Characterisation of Phosphofructokinase Purified from *Setaria cervi*: A Bovine Filarial Parasite

**DOI:** 10.4061/2011/939472

**Published:** 2011-09-15

**Authors:** Bechan Sharma

**Affiliations:** Department of Biochemistry, University of Allahabad, Allahabad 211002, India

## Abstract

Phosphofructokinase (PFK), a regulatory enzyme in glycolytic pathway, has been purified to electrophoretic homogeneity from adult female *Setaria cervi* and partially characterized. For this enzyme, the Lineweaver-Burk's double reciprocal plots of initial rates and D-fructose-6-phosphate (F-6-P) or Mg-ATP concentrations for varying values of cosubstrate concentration gave intersecting lines indicating that *K*_*m*_ values for F-6-P (1.05 mM) and ATP (3 *μ*M) were independent of each other. *S. cervi* PFK, when assayed at inhibitory concentration of ATP (>0.1 mM), exhibited sigmoidal behavior towards binding with F-6-P with a Hill coefficient (*n*) value equal to 1.8 and 1.7 at 1.0 and 0.33 mM ATP, respectively. D-fructose-1,6-diphosphate (FDP) competitively inhibited the filarial enzyme: *K*_*i*_ and Hill coefficient values being 0.18 *μ*M and 2.0, respectively. Phosphoenolpyruvate (PEP) also inhibited the enzyme competitively with the *K*_*i*_ value equal to 0.8 mM. The Hill coefficient values (>1.5) for F-6-P (at inhibitory concentration of ATP) and FDP suggested its positive cooperative kinetics towards F-6-P and FDP, showing presence of more than one binding sites for these molecules in enzyme protein and allosteric nature of the filarial enzyme. The product inhibition studies gave us the only compatible mechanism of random addition process with a probable orientation of substrates and products on the enzyme surface.

## 1. Introduction

Phosphofructokinase (ATP: D-fructose-6-phosphotrans-ferase, EC 2.7.1.11, PFK) is an enzyme of prime importance in the regulation of glycolytic flux in both the mammalian [1–5] and parasites [6–8], for example nematodes such as filarial worms *Setaria cervi *[9–13] and *Dirofilaria immitis* [14], *Ascaris suum* [15], malaria parasite *Plasmodium berghei* [16], intestinal parasite *Entamoeba *histolytica [17], liver fluke *Fasciola hepatica* [18], and a few others including *Toxoplasma gondii* [19], *Trypanosoma *cruzi [20], and *Trypanosoma brucei* [21]. The filarial worms studied so far employ predominantly anaerobic metabolism of carbohydrate (glycogen and/glucose) as a major source of energy [22]. However, they do not catalyze the complete oxidation of the substrate to CO_2_ and reduced organic acids as end product of the metabolism. The filarial nematodes utilize limited quantity of oxygen, when available and possess rudimentary and unusual electron transport chains that catalyze limited terminal oxidation with generation of little energy [23].

The kinetic and regulatory properties of the purified enzyme have been extensively studied in several mammalian systems, plants, and bacteria [24, 25]. In most of the cases, it is shown that PFK regulates glucose metabolism. Because of the multiplicity of modifiers, PFK has served as a model in studies of allosteric regulation of enzymes. The enzyme activity appears to be modulated to meet the metabolic needs of the cell, with the metabolites serving as intracellular indicators [26]. However, very little attention has been paid towards the systematic studies on the nature and properties of this enzyme from the filarial parasites.

*Setaria cervi*, a bovine filarial parasite, dwelling in the intraperitoneal cavity and lymphatics, possesses similarities with the human filarial worms in many ways such as nocturnal periodicity, antigenic composition, and the metabolic pathways. This parasite relies mainly on glycolysis for deriving energy [27]. It contains comparatively low levels of PFK and is thus highly vulnerable to the inhibitors of the glycolytic enzymes. PFK from *S. cervi* has been purified to electrophoretic homogeneity and some of its molecular properties have been studied [22]. It has been shown that this enzyme possess two different pH optima depending on ATP concentrations, the values being 8.0 at low (0.1 mM) concentration which decreases to pH 7.4 at high ATP (>0.1 mM) concentration. These results indicated that the activity of filarial PFK was possibly under regulation of ATP levels [28].

The present paper describes certain kinetic properties of PFK purified from adult female filarial worm, *S. cervi*, at low and high concentrations of ATP. It is shown that the molecular characteristics and reaction mechanisms of filarial PFK as deduced using steady state kinetic method differ radically from those of the complex vertebrates, thereby suggesting the adaptation of the filarial parasite in the anaerobic habitat.

## 2. Materials and Methods

### 2.1. Materials

#### 2.1.1. Parasite

Motile adult female worms were collected from the peritoneal folds of freshly slaughtered buffaloes at a local abattoir and brought to the laboratory in glucose saline. Worms were thoroughly washed three to four times with lukewarm isotonic saline. The fresh or the frozen worms at −20°C for a week were utilized for this study.

#### 2.1.2. Chemicals/Biochemicals

D-fructose-6-phosphate (F-6-P), adenosine-3′, 5′-triphosphate (ATP), *α*-glycerophosphate dehydrogenase (GDH), D-fructose-1,6-diphosphate (FDP), triosephosphate isomerase (TPI), aldolase, and phosphoenolpyruvate (PEP) were purchased from Sigma Chemical Co., USA. Nicotinamide adenine dinucleotide reduced (NADH) was obtained from CSIR Centre of Biochemical Technology, New Delhi. Other reagents used were analytical grade.

### 2.2. Methods

#### 2.2.1. Purification of *S. cervi* PFK

PFK from adult female *S. cervi* has been purified to electrophoretic homogeneity using very simple procedures and the activity has been stabilized using suitable reagents [22]. This purified enzyme preparation was used in the present study.

#### 2.2.2. Enzyme Assay

*S. cervi* PFK was assayed using an enzyme coupled reaction method described by Racker (1947) [29] with slight modification as described in [22]. In this method, we measured the formation of the D-Fructose-1,6-diphosphate (FDP) using aldolase, TPI, GDH, and NADH. The reaction mixture (3 mL) contained Tris HCl buffer (50 mM, pH 8.0), F-6-P (3.3 mM), ATP (0.1 mM), MgCl_2_ (3.3 mM), NADH (0.04 mM), GDH (0.66 Units/mL), TPI (5.6 Units/mL), aldolase (0.21 Units/mL), and suitable amount of enzyme protein (10–20 *μ*g). The reaction was always started by adding substrate to the reaction mixture and the change in absorbance (oxidation of NADH to NAD^+^) after every 30 sec interval was measured spectrophotometrically at 340 nm. The three auxiliary enzymes such as aldolase, GDH, and TPI were added in excess so that the overall reaction was governed by PFK activity present in the assay mixture. The concentration of Mg^2+^ was kept higher than that of ATP (unless stated otherwise) for generating Mg-ATP complex (the substrate for the enzyme) and avoiding presence of free ATP molecules, which are known to be inhibitory in nature to PFK from other sources [30].

#### 2.2.3. Determination of *K*_*m*_ and *K*_cat_ Values for Substrates of *S. cervi* PFK

The mechanism of enzyme action was investigated using steady state kinetic methods [31] and it was measured in the direction of formation of FDP (from F-6-P, ATP, Mg^2+^ ion) in the presence of NADH and nonratelimiting concentrations of auxiliary enzymes (aldolase, TPI and GDH). The *K*_*m*_ and *K*_cat_ values of F-6-P and ATP were determined by studying the rate of PFK catalyzed reaction at different concentrations of one substrate keeping the concentration of the second substrate constant. The *K*_cat_ values were based on a subunit mass of 90 kDa [22].

#### 2.2.4. Determination of *K*_*m*_ Value of MgCl_2_ for *S. cervi* PFK

The *S. cervi* PFK was assayed at varying concentrations of MgCl_2_ (0.05–7mM) keeping concentrations of ATP (0.1 mM) and F-6-P (3.3 mM) constant. The *K*_*m*_ value of MgCl_2_ was computed from equation deduced from Lineweaver-Burk's double reciprocal plot.

#### 2.2.5. Effect of Divalent Cations

To determine the effect of divalent metal ions such as Mg^2+^, Ca^2+^, Mn^2+^, Co^2+^, Cd^2+^, and Ba^2+^ on *S. cervi* PFK catalyzed reaction, assays were carried out under standard conditions (0.1 mM ATP, 3.3 mM F-6-P). The metal ion concentration in each case was 3.3 mM. The rate observed at 3.3 mM MgCl_2_ has been taken to be 100%.

#### 2.2.6. Product Inhibition Studies

The inhibition of *S. cervi* PFK by fructose-1,6-diphosphate (FDP) and phosphoenol pyruvate (PEP) was determined by assaying the enzyme at varying concentrations of F-6-P (0.5–5.0 mM) in presence and absence of fixed concentrations of FDP or PEP keeping the concentrations of ATP (0.1 mM) and Mg^2+^ (3.3 mM) constant. While studying the effect of FDP on activity of filarial PFK, suitable control was used (employing the reaction mixture containing FDP in the presence and absence of enzyme protein), which was deduced from experimental one to record the actual effect of FDP on activity of filarial PFK. Inhibition constants were calculated using the equation deduced from Lineweaver-Burk's double reciprocal plot.

#### 2.2.7. Other Analytical Methods

The mechanism of enzyme action was investigated using steady state kinetic methods and it was measured in the direction of formation of FDP (from F-6-P, ATP, Mg^2+^ ion) in the presence of NADH and nonratelimiting concentrations of auxiliary enzymes (aldolase, TPI and GDH). *K*_*m*_ values of F-6-P and ATP were determined by studying the rate of PFK catalyzed reaction at different concentrations of one substrate keeping the concentration of the second substrate constant.

## 3. Results

### 3.1. Effect of D-Fructose-6-Phosphate and ATP on the Phosphofructokinase Activity

**Figure 1:**
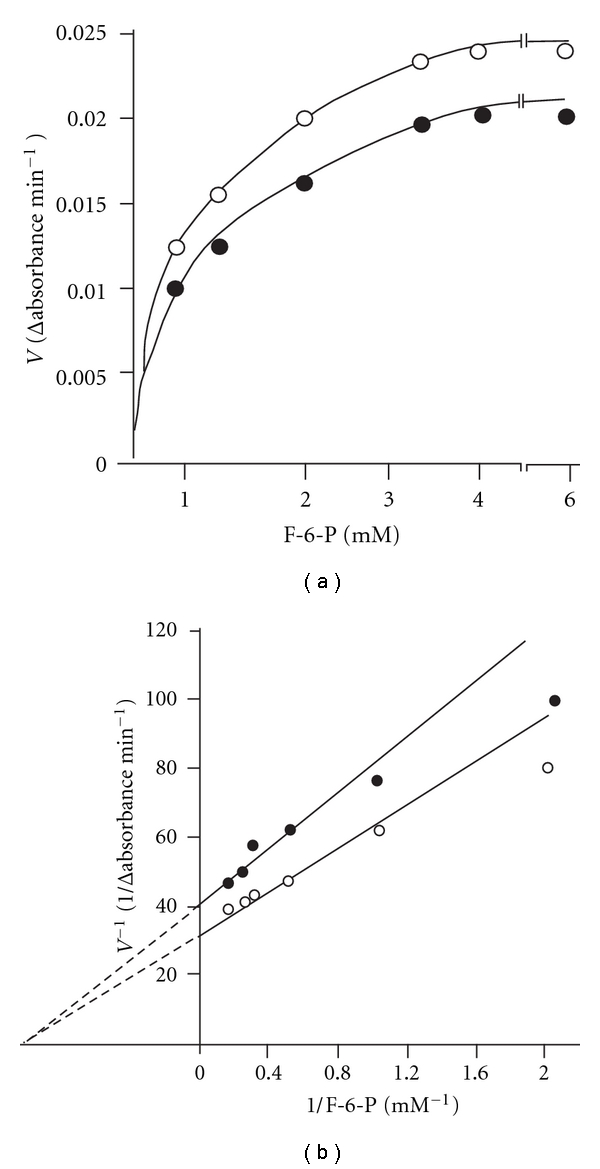
Effect of F-6-P concentration on the rate of *S. cervi *PFK catalyzed reaction. ATP concentration was 50 (○) and 100 (•) *μ*M and Mg^2+^ concentration was 3.3 mM. Enzyme concentration was 6.6 *μ*g/mL. Other conditions were same as in standard enzyme assay. (b) Double reciprocal plot of the data of (a). ATP concentrations were 50 (○) and 100 (•) *μ*M.

**Figure 2:**
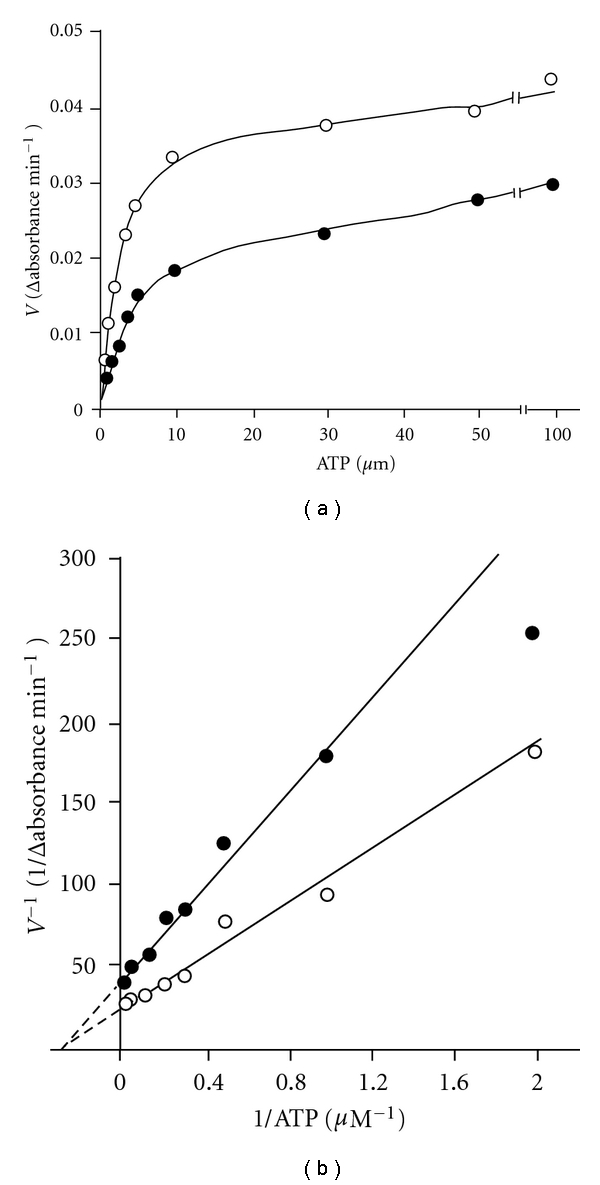
Effect of ATP concentration on the rate of PFK catalyzed reaction. F-6-P concentration was 1.6 (○) and 3.3 (•) mM and Mg^2+^ concentration was 3.3 mM. Enzyme concentration was 10 *μ*g/mL. Other conditions were same as in standard enzyme assay. (b) Double reciprocal plot of the data of (a). F-6-P concentrations were 1.6 (Δ) and 3.3 (▲) mM.

In order to study the effect of F-6-P, the enzyme was assayed using varying concentrations of F-6-P at two different concentrations of ATP (50 and 100 *μ*M). Similarly, the effect of ATP was studied by assaying the enzyme activity using varying concentrations of ATP and two fixed concentrations of F-6-P (1.7 and 3.3 mM). The *K*_*m*_ and V_max_ values were computed from the Lineweaver-Burk's double reciprocal plots. The results are shown in Figures 1 and 2. In each case the enzyme exhibited hyperbolic saturation curves. The double reciprocal plots as shown in Figures 1(b) and 2(b) gave sets of straight lines intersecting at common points on the negative abscissa from which the *K*_*m*_ values of 1.05 mm and 3 *μ*M were obtained for F-6-P and Mg-ATP^2−^, respectively. These observations further suggested that the *K*_*m*_ values of Mg-ATP^2−^ and F-6-P were not affected by varying the concentrations of other reactant (noncompetitive behavior). This result is consistent with the random order of attachment of the substrates to the enzyme. The low *K*_m_ value of for ATP (3 *μ*M) is noteworthy. Further the Mg^2+^ was always quite high (3.3 mM), so that all ATP must have existed as Mg-ATP complex. Thus the results suggested that *S. cervi* PFK had high affinity for Mg-ATP complex. However, the *S. cervi *enzyme was found to be highly specific for F-6-P: at 3.3 mM the relative activities with D-glucose-6-phosphate and D-glucose-1-phosphate were 10.7 and 3.2%, respectively, of the rate with F-6-P. The *K*_cat_ value was found to be 320 ± 15 sec^−1^.

### 3.2. Effect of Higher Concentrations of ATP on Filarial PFK Catalyzed Reaction

**Figure 3:**
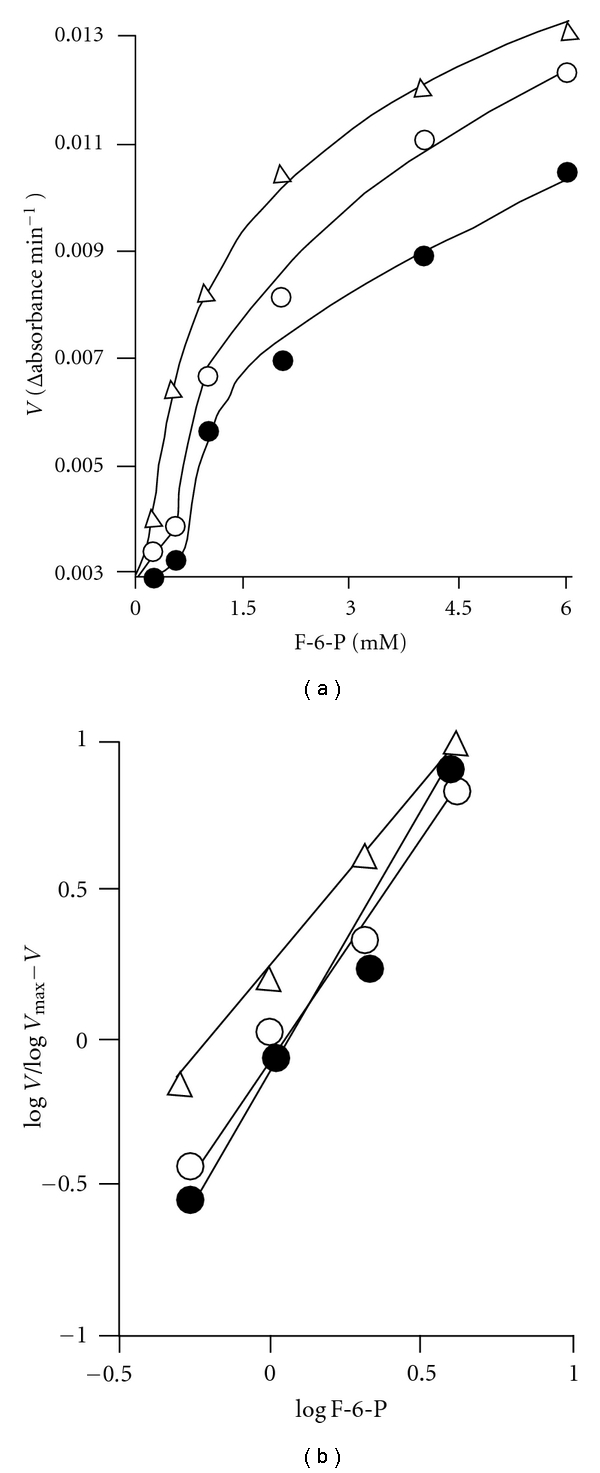
Effect of F-6-P concentration on the rate of *S. cervi* PFK catalyzed reaction at different fixed and high concentration of ATP. ATP concentration was 0.1 (Δ), 0.33 (○), and 1.0 (•) mM and Mg^2+^ concentration was 3.3 mM. Enzyme concentration was 3.3 *μ*g/mL. Other conditions were same as in standard enzyme assay. Inset. Hill plot of the data of (a) at 0.2–6.0 mM F-6-P concentrations. ATP concentrations were 0.1 (Δ), 0.33 (○), and 1.0 (•).

At higher concentration of ATP (330 and 1000 *μ*M) and 3.3 mM Mg^2+^ concentration, the reaction rate versus F-6-P concentration curve was found to be sigmoidal in shape (Figure 3). The sigmoidicity in these data appears to be function of ATP concentration. Correspondingly, slope of the Hill plot (*n*) of the data (Figure 3(b)) was found to depend on ATP concentration. Its values were found to be 1.8, 1.7, and 1.1 at 1.0, 0.33 and 0.1 mM ATP concentrations, respectively.

**Figure 4:**
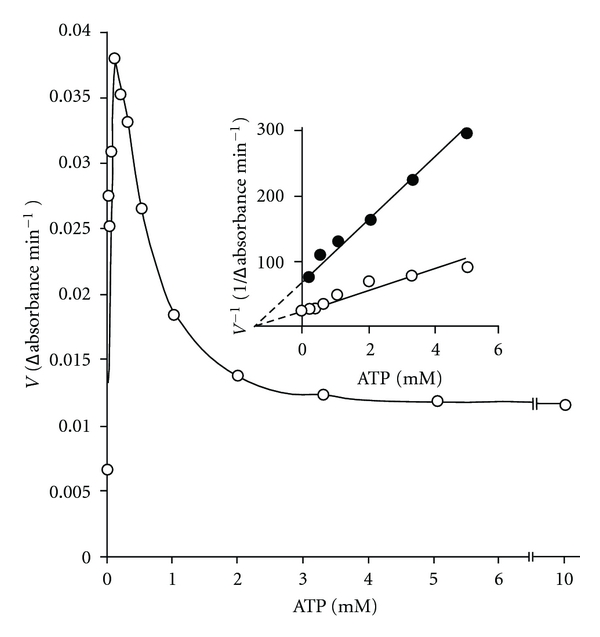
Effect of ATP concentration on the rate of PFK catalyzed reaction at fixed concentration of F-6-P and Mg^2+^ (3.3 mM each). Inset shows the plot obtained on assaying the enzyme at variable inhibitory (>0.1 mM) concentration of ATP at two fixed concentrations of F-6-P such as 1.6 (•) and 3.3 (○) mM. Mg^2+^ concentration was constant (3.3 mM). Enzyme concentration was 10 *μ*g/mL. Other conditions were same as in standard enzyme assay.

It may be noted that concentrations of ATP, which gave rise to sigmoidicity in F-6-P saturation curve in Figure 3 were higher than those used in Figures 1 and 2, where simple hyperbolic saturation curves were obtained for both the substrates. It became necessary, therefore, to extend the experiments for the effect of ATP to higher concentration ranges. Results of such an experiment carried out at a single fixed and high F-6-P concentration (3.3 mM) are shown in Figure 4. The concentration of Mg^2+^ was held high and constant (3.3 mM). Rate of reaction increased progressively up to 0.10 mM ATP concentration, beyond which a sharp decline was observed suggesting inhibition of the enzyme at higher ATP concentrations. The plots of the data obtained at high and inhibitory ATP concentrations (1/rate of reaction versus ATP) and at two fixed F-6-P concentrations, namely, 1.7 and 3.33 mM (Figure 4; inset) showed that the enzyme had an allosteric site for ATP with low affinity. The substrate inhibition constant (*K*_*i*_^*s*^) value was 1.2 ± 0.1 mM. 

### 3.3. Concentration of Mg^2+^ Ion Is Limiting for Filarial PFK Activity

**Figure 5:**
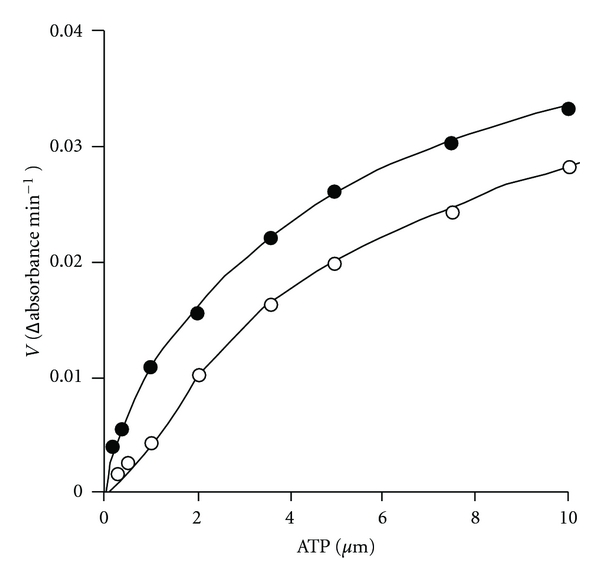
Effect of Mg^2+^ and ATP concentrations on the rate of PFK-catalyzed reaction. For (○), Mg^2+^ ion concentration was equal to and varied together with that of ATP. For (•), Mg^2+^ ion concentration was equal to 3.33 mM. F-6-P concentration in each case was 3.33 mM. Enzyme concentration was 10 *μ*g/mL. Other conditions were same as in standard enzyme assay.

In a separate set of experiments, concentration of F-6-P was maintained constant at 3.3 mM and concentrations of Mg^2+^ and ATP were varied together and kept equal to each other. The data showed an apparent sigmoidicity when rate of reaction was plotted against [ATP]. The results indicate that sigmoidicity is observed at low values of Mg^2+^ and ATP concentrations (Figure 5). Under these conditions, the dissociation of Mg-ATP complex will be significant

(1) $${\left(\text{Mg-ATP}\right)}^{2-}⟷\;\text{M}{\text{g}}^{2}+\;+\;\text{AT}{\text{P}}^{4-}.$$

At lower concentrations, the concentration of complex will be less than that of Mg^2+^and ATP added. If Mg-ATP complex (rather than free ATP and Mg^2+^) is the real substrate, the observed rate of reaction will be lower than expected. This will make the curve sigmoidal. Correspondingly, when concentration of Mg^2+^ is kept high and constant (3.3 mM), all available ATP will exist as Mg-ATP complex. Under these conditions rate versus ATP concentration plot is no longer sigmoidal but gives usual hyperbolic curve (Figure 5).

### 3.4. Presence of Excess Concentration of Cations, Specifically Mg^2+^ Ions, Is Required by Filarial PFK

**Figure 6:**
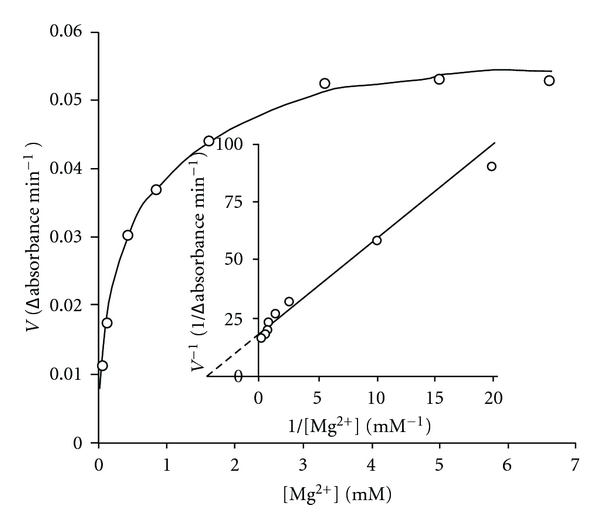
Effect of Mg^2+^ concentration on the rate of PFK-catalyzed reaction. Concentrations of F-6-P and ATP were 3.33 and 0.1 mM, respectively. Enzyme concentration was 10 *μ*g/mL. Other conditions were same as in standard enzyme assay. Inset shows the double reciprocal plot of the data.

The effect of divalent metal ions (Mg^2+^, Ca^2+^, Mn^2+^, Co^2+^, Cd^2+^, and Ba^2+^) on *S. cervi* PFK catalyzed reaction was studied in order to find out the specificity and preferential utilization of metals for optimal activity. The *S. cervi* PFK exhibited very low (2%) activity when Mg^2+^ was omitted from the assay solution (Table 1). The filarial PFK activity exhibits Mg^2+^ concentration dependence (Figure 6). The *K*_m_ of Mg^2+^ was found to be 0.23 ± 0.02 mM. Mn^2+^, Ca^2+^ and Co^2+^ (each at 3.3 mM concentration) showed 36, 11 and 5% of the activity observed with Mg^2+^. The other metal ions tested, Cd^2+^ and Ba^2+^ did not show any activity. 

### 3.5. Effect of Products (D-Fructose-1,6-Diphosphate (FDP)) and Phosphoenolpyruvate (PEP) on the Activity of *S. cervi* Phosphofructokinase

**Figure 7:**
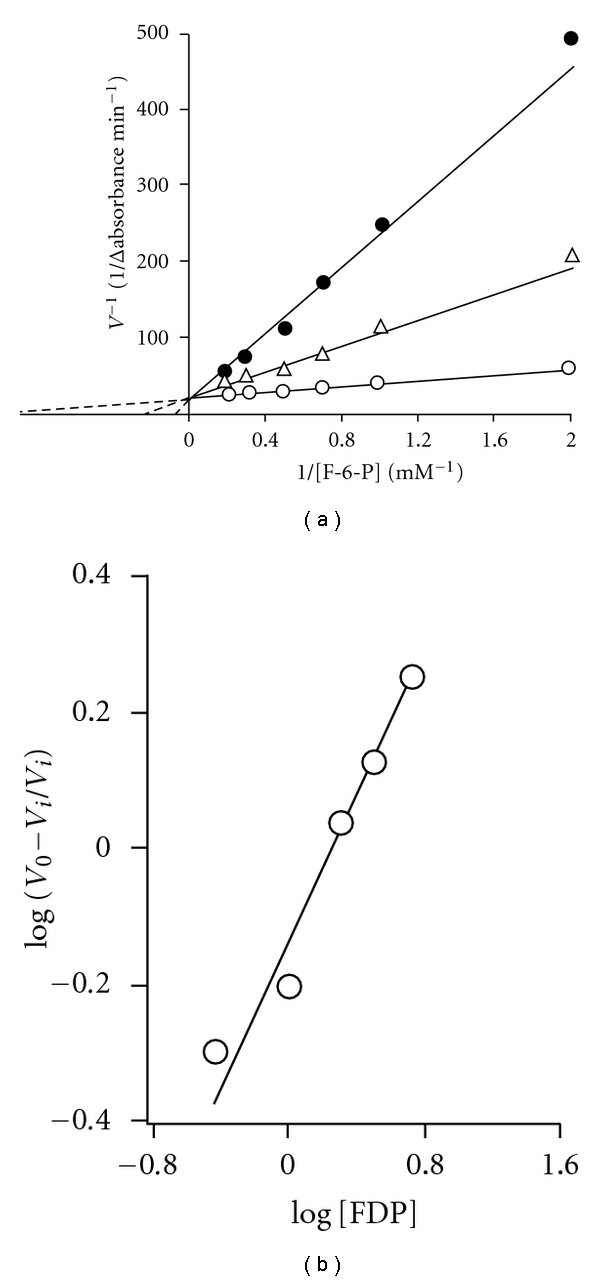
(a) Effect of FDP on the rate of PFK catalyzed reaction. FDP concentrations were nil (○), 1.0 (Δ), and 2.0 (▲) *μ*M. Mg^2+^ concentration was constant (3.3 mM). The concentration of ATP was 0.1 mM. Enzyme concentration was 10 *μ*g/mL. Other conditions were same as in standard enzyme assay. (b) Hill plot of the data of the effect of FDP on the rate of PFK catalyzed reaction. The concentrations of F-6-P, ATP, and Mg^2+^ were 3.3, 0.1, and 3.3 mM, respectively. Enzyme concentration was 6.6 *μ*g/mL. *V*_*i*_ and *V*_0_ are the rates of reaction in the presence and absence of FDP. Other conditions were same as in standard enzyme assay.

**Figure 8:**
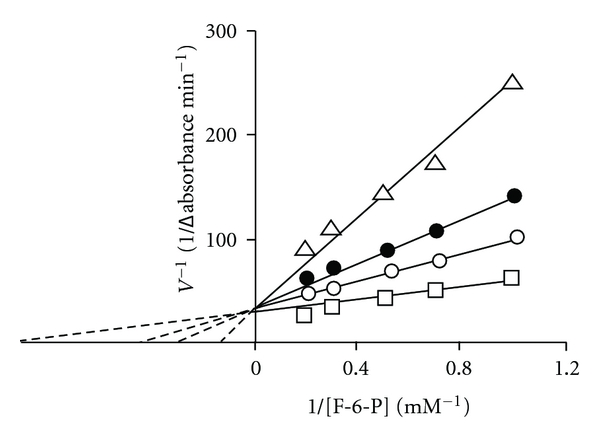
Effect of PEP on the rate of PFK-catalyzed reaction. PEP concentrations were nil (□), 1.0 (○), 2.0 (•), and 5.0 (Δ) mM. Mg^2+^ and ATP concentrations were 3.3 and 0.1 mM, respectively. Enzyme concentration was 10 *μ*g/mL. Other conditions were same as in standard enzyme assay.

The product inhibition studies were carried out using F-6-P as a variable substrate and near saturating (0.1 mM) concentration of cosubstrate (Mg-ATP). The results showed that the inhibition of PFK activity by FDP was competitive in nature (Figure 7(a)) with respect to F-6-P; the *K*_*i*_ value as computed from the Lineweaver-Burk's double reciprocal plot was found to be 0.18 *μ*M. A Hill plot of the data at several FDP concentrations and a fixed F-6-P concentration (Figure 7(b)) showed a slope equal to 2.0, suggesting cooperativity in the binding of FDP. PEP, another metabolite in glycolysis, also showed a cooperative type of inhibition with respective F-6-P (Figure 8) and its *K*_i_ value was found to be 0.8 mM.

## 4. Discussion

### 4.1. The Crude and Purified Preparations of *S. cervi* PFK Have Same *K*_*m*_ for F-6-P

Earlier works from this laboratory have demonstrated the existence of PFK in *S. cervi* comparatively in lesser amount. This enzyme has been purified and some of its molecular characteristics were studied [22]. The *K*_*m*_ of F-6-P for *S. cervi* PFK and its inhibition by high concentration of ATP with crude preparation [28] have been further confirmed with the purified enzyme [22]. This agreement suggests that no kinetically relevant proteolytic modifications occurred during purification.

### 4.2. *S. cervi* PFK Exhibits Specificity in Having More Affinity to Mg-ATP Complex Than F-6-P

The much lower *K*_*m*_ value of Mg-ATP (3 *μ*M) as compared to that of F-6-P (1.05 mM) for *S. cervi* enzyme at pH 8.0, suggests higher affinity of the enzyme to ATP than F-6-P. Similar wide differences in *K*_*m*_ values of Mg-ATP and F-6-P were also observed with the enzyme isolated from other parasites such as *M. expansa* [32], *A. suum* [15] *T. brucei *[21], and *T. cruzi* [20] whereas the enzyme from vertebrate tissues exhibited very less difference between the *K*_*m*_ values of Mg-ATP and F-6-P [33]. The binding of the substrate Mg-ATP to PFKs from different origins shows a hyperbolic dependence with half saturation values in the range of 10–100 *μ*M in most of the cases. However, it was quite low (3 *μ*M) for PFK from *S. cervi.*

### 4.3. The Affinity of *S. cervi* PFK for One Substrate Is Independent of the Concentration of the Cosubstrate

The complex allosteric interactions of substrate and effectors with PFK hampered the study of the reaction mechanism of this enzyme from most of the sources. The study of the reaction mechanism of *S. cervi* PFK was carried out under optimal conditions using steady state kinetic methods [34, 35]. The intersecting lines of Lineweaver-Burk's double reciprocal plots (Figures 1 and 2) clearly indicated that the affinity of the enzyme for one substrate was independent of the concentration of the cosubstrate. Similar results were reported for the enzyme of *T. cruzi* [20] However, for the enzyme in *M. expansa* and *T. brucei* [36], the *K*_*m*_ value of Mg-ATP^2-^ has been shown to increase at higher F-6-P concentration. The *K*_cat_ value (320 ± 15 sec^−1^) for F-6-P as recorded for *S. cervi* PFK in present investigation was found to be similar to that from *E. histolytica* (344 sec^−1^) [37] and about five times higher than that of *T. brucei* (61 sec^−1^) [38].

### 4.4. ATP Regulates Activity of *S. cervi* PFK

The purified enzyme from *S. cervi* displayed regulatory properties which confirmed previous reports from this laboratory with a cytosolic preparation particularly its inhibition by higher concentration of ATP (>0.1 mM), which is the basic regulatory property of PFK in vertebrates, bacteria, and a few plants responsible for Pasteur effect [12]. The steady state kinetic results, as stated above, were obtained when ATP concentration was equal to or less than 0.1 mM. The rate of reaction increased to maximum with ATP concentration up to 0.1 mM (in presence of 3.3 mM F-6-P and 3.3 mM Mg^2+^) (Figure 4). At higher ATP concentration, a lowering of catalyzed rate was observed (inhibition by excess ATP). The enzyme from other mammalian [39] and parasite sources [18] required more ATP for optimal activity. The corresponding optimum ATP concentrations for the monkey liver [40] and rat small intestine enzymes [41] were found to be 0.4 and 1.0 mM, respectively. The enzymes from *A*. *suum* [15] and *F. hepatica* [42] require 50–200 and 200 *μ*M ATP concentrations, respectively, for showing maximal activity.

The kinetics of ATP and F-6-P saturation for *S. cervi* enzyme are almost identical to those reported in *D. immitis* and *A. suum* [43]. The inhibitory action of ATP was reported for the first time with rabbit muscle PFK and the inhibition was attributed to isosteric competition with the true substrate, that is, Mg-ATP complex. However, with the increase in F-6-P concentration, the inhibitory action of ATP was reduced suggesting that the ability to demonstrate the regulatory effects of either substrate is a function of relative concentration of other substrate.

### 4.5. Allosteric Regulation of *S. cervi* PFK

The inhibition of rabbit muscle PFK by ATP has been reported to be pH dependent [44–46]. In contrast to these findings, the enzyme from *T. cruzi* was not inhibited by ATP even at 1.0 mM concentration at pH 7.4. The inhibition pattern (noncompetitive behavior) of *S. cervi* PFK by ATP is in close agreement with that of pig spleen enzyme. The filarial PFK showed sigmoidicity in saturation with respect to F-6-P when assayed at inhibitory concentration of ATP with Hill coefficient (*n*) values found to be 1.8. The complex kinetics of pure *S. cervi* enzyme towards F-6-P (Figure 3) has also been reported for analogous enzyme isolated from mammalian and parasite sources. It has been explained assuming more than two binding sites per catalytic unit with cooperative interactions, by the presence of two or more isozymes with different kinetic properties or by changes in the aggregation state and the activity of the enzyme induced by the combination of protein and substrate (particularly F-6-P) concentrations.

### 4.6. Mg-ATP Complex Is the Real Substrate of *S. cervi* PFK

In all the experiments described above, Mg^2+^ concentration was maintained high and constant (3.3 mM). If Mg^2+^ and ATP concentrations are maintained equal and varied together keeping F-6-P concentration high and constant (3.3 mM), some deviation from the hyperbolic saturation is observed. The deviation is observed when both Mg^2+^ and ATP concentrations are low (2 *μ*M). The rate versus (Mg^2+^) = (ATP) curve has a sigmoidal shape. At low Mg^2+^ and ATP concentrations, dissociation of Mg-ATP complex into free Mg^2+^ and ATP will be considerable and significant. At higher concentrations, on the other hand, Mg^2+^ and ATP will exist mostly in the form of Mg-ATP complex. If the latter (rather than free ATP) is the real substrate for enzyme, its concentration will decrease sharply (and disproportionately) at lower concentrations. Consequently, lower rates of reaction will be obtained than those calculated if all ATP + Mg^2+^ existed as Mg-ATP complex. This will generate an apparent sigmoidicity without there being any cooperativity in binding of Mg-ATP to enzyme. Further, free ATP is expected to bind better to the allosteric or inhibitory site of enzyme. This will enhance the sigmoidicity. Thus our data suggest that Mg-ATP is the real substrate of *S. cervi* PFK.

The complex kinetics of pure *S. cervi* enzyme towards F-6-P (Figure 3) has also been reported for analogous enzyme isolated from many other sources. It has been explained assuming more than two binding sites per catalytic unit with cooperative interactions, by changes in activity of the enzyme induced by the combination of protein and substrate (particularly F-6-P) concentrations.

In summary, three types of steady state kinetic results have been described. In one set of experiments, noninhibitory concentrations of ATP (≤0.1 mM) and high Mg^2+^ concentration (3.3 mM) were used and hyperbolic saturation was observed for F-6-P as well as ATP. Their *K*_*m*_ values (1.05 mM and 3 *μ*M, respectively) were independent of the concentration of other substrate. In second set, when inhibitory concentrations of ATP (1.0 mM or above) were employed at high Mg^2+^ concentration (3.3 mM), F-6-P binding shows sigmoidicity with a Hill coefficient (*n*) equal to 1.8. In third set, non-inhibitory concentrations of ATP were employed, but Mg^2+^ and ATP concentrations were held equal and varied together. Concentration of F-6-P was held constant at 3.3 mM. An apparent sigmoidicity was observed in the plot of rate versus ATP concentration. This is explained on the basis of assumption that Mg-ATP complex, rather than free ATP is the real substrate for this enzyme.

### 4.7. *S. cervi* PFK Preferentially Utilizes Mg^2+^ for Its Optimal Activity

The results of the effect of some divalent cations on activity of *S. cervi* PFK suggested that this enzyme possess a wide specificity for metal ions like analogous enzyme from mammalian sources. However, unlike other systems, filarial PFK exhibits preferential utilization of Mg^2+^ for its optimal activity. The *K*_*m*_ of Mg^2+^ for *S. cervi* PFK (0.23 ± 0.02 mM) is nearly five times lower than the value reported for rabbit brain (1.2 mM), and very close to value for *T. brucei* (0.294 mM) [21].

### 4.8. Feedback Inhibition of *S. cervi* PFK

The product inhibition studies were carried out using F-6-P as a variable substrate (because of its simple Michaelis-Menten kinetics at all concentrations) and near saturating (0.1 mM) concentration of Mg-ATP. *S. cervi* PFK was found to be inhibited by FDP and PEP in a competitive manner, the former was inhibitory at micromolar, while the latter was effective at millimolar concentrations. The enzymes from *T. brucei* and *T. cruzi *were also inhibited by FDP, while that from *A. suum* and epimastigotes of *T. cruzi* remained unaffected on treatment with FDP. The enzyme from mammalian sources, however, was significantly activated by FDP. PEP is well-known inhibitor of PFK from mammalian systems and parasites. It has been reported that PEP acts as an allosteric inhibitor of PFK from *T. brucei*. In present investigation, the *K*_*i*_ value of PEP for *S. cervi* PFK was half of the value reported for the pig spleen enzyme. PEP, however, has been shown to activate the enzyme from malaria parasite [16]. 

The product inhibition patterns displayed by *S. cervi* PFK (Figures 5 and 6) are only compatible with random addition process with the probable orientation of the substrate and product on the enzyme surface in which random entry of substrates produces a central complex which isomerizes and releases the products in random fashion. Again the proposed mechanism for *S. cervi* PFK is markedly different from those reported for the analogous enzyme from many other sources: the results of the studies on the mechanism of mammalian PFK has been shown to exhibit both random and ordered sequential bi-bi process [39]; bacterial enzyme possesses an ordered bi-bi mechanism [24], while the enzyme from the yeast and slime mold favors an apparent ping-pong bi-bi. On the other hand, the proposed reaction mechanism of random order of the entry of the substrates to the active site of *S. cervi* enzyme has been reported mainly for allosteric PFK, which is much different from those reported for nonallosteric analogous enzyme from certain other sources [47, 48].

## 5. Conclusion

In conclusion, the filarial enzyme shows allosteric properties similar to PFK from various sources; it is inhibited by ATP and exhibits two different pH optima depending on the ATP concentrations but it differs with allosteric regulation. The *S. cervi* follows a random mechanism of substrate binding. The filarial enzyme appears to be parasite specific which differs in molecular and kinetic properties from that of the mammalian sources. These differences may be attributed with an adaptation of this parasite to an anaerobic utilization of glucose. Further, the unique properties of *S. cervi* enzyme could be exploited for an effective and parasite-specific antifilarial drug design.

## Figures and Tables

**Table 1:** Effect of some divalent metal ions on the activity of PFK from *S. cervi. *

Salt	Relative activity
MgCl_2_	100
CaCl_2_	36
MnCl_2_	11
CoCl_2_	5
CdCl_2_	0
BaCl_2_	0
No salt	2

Assays were carried out at optimal concentration of F-6-P (3.3 mM) and ATP (0.1 mM) following the procedure as described in Section 2. The metal ion concentration in each case was 3.3 mM. The rate of reaction determined at 3.3 mM MgCl_2_ is considered to be 100%.
